# Cirsiliol regulates mitophagy in colon cancer cells via STAT3 signaling

**DOI:** 10.1186/s12935-022-02732-6

**Published:** 2022-10-07

**Authors:** Tao Jiang, Lulu Peng, Qian Wang, Bingyu Huang, Dewei Peng, Lintong Men, Yue Jiang, Mengying Zhu, Moran Wang, Li Lin, Jiagao Lv, Sheng Li

**Affiliations:** 1 Department of Geriatrics, Tongji Hospital, Tongji Medical College, Huazhong University of Science and Technology, Wuhan, China; 2 Division of Cardiology, Department of Internal Medicine, Tongji Hospital, Tongji Medical College, Huazhong University of Science and Technology, Jiefang Avenue 1095, P.O. Box 430030, Wuhan, People’s Republic of China

**Keywords:** Colon cancer, Cirsiliol, STAT3, Mitophagy, ROS

## Abstract

**Background:**

Mitophagy is a type of selective autophagy for dysfunctional mitochondria and plays a key role in tumorigenesis and cancer progression. However, whether mitophagy plays a role in colon cancer remains unclear. Cirsiliol is a natural product and has been found to exert anti-cancer effects in multiple tumors. The effects of cirsiliol in the tumorigenesis and progression of colon cancer remain unknown.

**Methods:**

CCK8 assay, plate cloning assay, and cell scratch assay were performed to determine cell viability, colony formation, and wound healing abilities of HCT116 and SW480 cells. JC-1 staining, H2DCFDA staining, and Mito-Tracker Red staining were carried out to evaluate mitochondrial membrane potential (Δψm), intracellular reactive oxygen species (ROS) level, and mitochondrial morphology. Molecular docking technology was utilized to predict interaction of cirsiliol and signal transducer and activator of transcription 3 (STAT3). Immunofluorescence staining was used to measure nuclear translocation of STAT3. The protein levels of phosphorylated STAT3 (Y705), total STAT3, and mitophagy proteins were detected by western blot.

**Results:**

In this study, we first found that cirsiliol inhibited cell viability, colony formation, and wound healing abilities of HCT116 and SW480 colon cancer cells. Moreover, cirsiliol suppressed Δψm, increased ROS production, and disrupted mitochondrial morphology via inhibiting the levels of mitophagy proteins including PINK1, Parkin, BNIP3, and FUNDC1. Application of mitophagy activator improved the levels of mitophagy-related proteins, and ameliorated Δψm and ROS levels. According to the result of molecular docking, we found that cirsiliol potentially bound to the SH2 domain of STAT3, the key domain for the functional activation of STAT3. Moreover, it was found that cirsiliol inhibited constitutive and IL‑6‑induced STAT3 phosphorylation and nuclear translocation by western blot and immunofluorescence analysis. Comparing with cirsiliol group, we found that overexpression of STAT3 restored the expressions of mitophagy proteins.

**Conclusions:**

Cirsiliol targets STAT3 to inhibit colon cancer cell proliferation by regulating mitophagy.

## Background

Colon cancer (CC) represents the second most frequent malignant neoplasm and becomes the second most common cause of cancer-related deaths globally [1]. Based on the 2021 cancer statistics, 104,000 new individuals are diagnosed with colon cancer, and around 53,000 of these people die in the United States [2]. Currently, the combination of surgery treatment, chemotherapy, and radiotherapy is the major therapeutic approach for early or intermediate stage patients with localized CC [3]. However, the patients in the late stages have a poor prognosis with only about 10% 5-year overall survival rate, and more than 50% of patients with CC are diagnosed at an advanced stage [4]. Thus, it is necessary to determine the specific mechanisms of the tumorigenesis and progression of CC.

Since ancient times, natural plants and compounds are widely used as clinical drugs, because of its high effectiveness and low toxicity. To date, natural products or natural products-derived compounds have served as the major therapeutic approaches, which accounts for over 60% of the available anti-cancer drugs [5]. Flavonoids are widely distributed in plants and they are important biologically active natural polyphenols. They have been reported to have multiple functions, including anti-cancer, anti-inflammatory, anti-oxidation, and anti-virus [6]. Cirsiliol is a kind of flavonoids found in many plants such as Leonotis nepetifolia, Artemisia, and Salvia, and has anti-infective, anti-obesity and anti-fungal activities [7, 8]. In recent years, cirsiliol has been found to exhibit anti-carcinogenic effect against esophageal squamous cell carcinoma, non-small cell lung cancer and so on [9, 10]. However, it has not yet been clarified whether cirsiliol experts the potential anti-tumor activities against CC. In addition, the targets of cirsiliol have not been clearly elucidated.

Mitophagy is the cellular process of clearance of mitochondria to maintain mitochondria mass and function [11]. Mitophagy exerts an important role in tumorigenesis and tumor progression. Several studies show that mitophagy promotes tumor progression, such as hepatocellular carcinoma [12], breast cancer [13], and cervical cancer [14]. However, the concrete mechanisms underlying the role of mitophagy in CC is unknown.

The purpose of the present study is to investigate whether cirsiliol can suppress colon cancer growth and its possible mechanisms.

## Methods

### Human colon cancer cell lines and culture

Human colon cancer cell lines (HCT116 and SW480 cells) were purchased from ATCC and maintained in high-glucose DMEM (KeyGEN BioTECH, Nanjing, China) supplemented with 10% fetal bovine serum (Sijiqing Bioengineering Material Co., Ltd., Hangzhou, China) and 1% penicillin/streptomycin (Sangon, Shanghai, China) at 37 °C and 5% CO_2_.

### Compounds

Cirsiliol was from Yuanye Bio-Technology Company (B31684; Shanghai, China); The powder of cirsiliol was dissolved in dimethyl sulfoxide (DMSO) at a concentration of 10 mM. UMI-77 was purchased from MCE (HY-18628; Monmouth Junction, NJ, USA) and dissolved in DMSO at 10 mM concentration. IL-6 was purchased from PeproTech (200-06; Rocky Hill, NJ, USA).

### Cell viability assay

A CCK8 assay kit (HY-K0301; MCE, Monmouth Junction, NJ, USA) was used to determine the cell viability. Briefly, HCT116 and SW480 cells were counted with the cell counter (Nexcelom Biosciences, Lawrence, USA) and 5000 cells were seeded in 96-well plates in each well (six wells per group) for 12 h. Then cirsiliol (10, 20, and 40 μM) was treated to the cells for 24 h. After treatment, cells were incubated with 10% CCK8 for 2 h at 37 °C protected from light. The absorbance was measured at 450 nm.

### Colony formation assay

Cells were seeded in six-well plates and treated without or with cirsiliol (10, 20 μM) for 24 h. After drug treatment, the cells were stained with trypan blue (Promoter Biotechnology, Wuhan, China) and counted. Viable cells were planted in 10-cm dishes at 5 × 10^3^ density for 2 weeks. Then 4% paraformaldehyde was applied to fix cells. After that, crystal violet (0.5% crystal violet, 20% methanol) was used to count the cell colonies.

### Wound healing assay

HCT116 and SW480 cells were seeded in six-well plates and maintained in complete medium. When cells grew to a confluence of 100%, a sterile 10 µL pipette tip was applied to scratch the monolayer cells. Then medium containing various concentrations of cirsiliol (10 and 20 μM) or DMSO was used to incubate cells for 24 h. Pictures were taken at 0, 24, and 48 h using a MShot microscope (Wuhan, China) and the wound-healing percentage was calculated using Image J (Fiji) software.

### Western blot

After treatment, cells were lysed using pre-cooled RIPA buffer containing phosphatase inhibitor and protease inhibitor cocktail for 1 h on ice. The cell lysates were centrifuged at 12,000 rpm for 20 min, then supernatants were collected. Total protein concentration of each sample was measured using the BCA method. Equal amounts of total protein were loaded on Bis–Tris gel and separated by SDS-PAGE. Then protein on gels was transferred to PVDF membranes which were then incubated with primary antibody at 4 °C overnight, including p-STAT3 (Tyr705) (#9145, Cell Signaling Technology, CST, Danvers, MA, USA), STAT3 (#4904, CST), FUNDC1 (#49240, CST), BNIP3 (ab10433, Cambridge, MA, Abcam), NIX (#12396S, CST), PINK1 (ab23707, Abcam), Parkin (#4211, CST), and GAPDH (#2118, CST). HRP-conjugated goat anti-rabbit and anti-mouse IgGs and ultra high sensitivity ECL kit (HY-K1005; MCE, Monmouth Junction, NJ, USA) were applied to develop protein. Images were captured using ChemiScope6100 Imager and ChemiScope software (Clinx Science Instruments, Shanghai, China).

### Measurement of mitochondrial membrane potential (Δψm)

Δψm was measured using JC-1 dye (PJC-110; Promotor Biological Co., Ltd., Wuhan, China), as previously described [15]. Following treatment, cells were incubated with JC-1 at cell incubator for 30 min followed by one wash with PBS, then PBS was replaced with fresh culture medium. The fluorescence intensity of JC-1 aggregate (red) and JC-1 monomer (green) were determined with MShot fluorescence microscope (20× objective lens) (Wuhan, China). Δψm was represented as the ratio of JC-1 aggregate/JC-1 monomer.

### Assessment of reactive oxygen species (ROS)

ROS production was detected by H2DCFDA (HY-D0940; MCE, Monmouth Junction, NJ, USA) fluorescent dye. After treatment, cells were incubated with 10 μM H2DCFDA in a cell incubator for 30 min, then washed with prewarmed Hanks’ balanced salt solution (HBSS, C0219; Beyotime Biotechnology, Shanghai, China). Then 4% paraformaldehyde was applied to fix cells for 15 min. The fluorescence signal was measured with the confocal microscope (40× objective lens) (Nikon confocal microscope, C2 + , Nikon, Tokyo).

### Mitochondria staining and analysis of mitochondrial network morphology

Mitochondria staining was performed using Mito-Tracker Red CMXRos (C1035; Beyotime Biotechnology, Shanghai, China) [15]. Briefly, SW480 cells were incubated with Mito-Tracker Red (100 nM) for 30 min at a cell incubator, then washed with prewarmed HBSS. Then 4% paraformaldehyde solution was used to fix cells. The cells were observed under the confocal microscope (Nikon confocal microscope, C2+, Nikon, Tokyo) using a 100× oil immersion to view the mitochondrial network morphology of cells. The mitochondrial network morphology was assessed using the ImageJ macro tool MiNA. Three parameters were applied to evaluate the mitochondrial network morphology: percentage of network mitochondria, mean branch length, and mitochondrial footprint.

### Molecular docking

Protein structure of STAT3 was acquired from the Protein Data Bank (PDB, PDB ID: 1BG1) [16]. Then water molecules in STAT3 were removed and polar hydrogen atoms were added in Discovery Studio Visualizer (BIOVIA, Discovery Studio Visualizer, Version 16.1.0.15350, Dassault Systèmes, San Diego, USA). Then prepared PDB was imported to AutoDock Tools 1.5.6 (The Scripps Research Institute, La Jolla, CA, USA) [17], computed gasteiger charges, then saved as a PDBQT docking input file. Moreover, a grid box was added to surround the whole protein structure. The chemical structure of cirsiliol was obtained from SciFinder (http://scifinder.cas.org) and energy minimization of structures was conducted using Chem3D. Then molecular docking of cirsiliol on 1BG1 was carried out using AutoDock Vina [18]. Finally, the docking results were analyzed using the Discovery Studio 2016.

#### Immunofluorescence staining

HCT116 cells were grown on coverslips for 12 h and exposed to treatments. After treatment, cells were washed with PBS and then fixed by immersion in 4% paraformaldehyde for 15 min at room temperature. After rinsing with PBS, cells were permeabilized and blocked with 0.5% Triton-100 and 5% goat serum in PBS for 1 h at room temperature. Coverslips were then incubated with primary antibodies against STAT3 (#9139, CST) at 4 °C, and followed by incubation with goat anti-mouse Cy3 secondary antibody for 1 h. Finally, the DAPI-containing anti-fluorescence quencher was used to seal the glass slides. Slides were then examined under the confocal microscope (Nikon confocal microscope, C2+, Nikon, Tokyo) using a 100× oil immersion.

### Transfection

HCT116 cells were seeded in six-well plates for 12 h and transfected with the pcDNA 3.1-human STAT3 or pcDNA 3.1 empty plasmid (Genomeditech Co. Ltd, Shanghai, China) using lipo3000 (L3000015; Invitrogen, USA). HCT116 cells were then incubated with 20 μM cirsiliol for 24 h at 48 h post-transfection.

### Statistical analysis

IBM SPSS 26.0 and GraphPad Prism 8.0 were used for statistical analyses and picture drawing. Data from all the experiments were presented as mean ± SD. Statistical analysis was performed using one-way analysis of variance (ANOVA) with Bonferroni’ s post-hoc test. Image analysis were performed with Image J (Fiji) software. For all the statistical tests, p < 0.05 was considered statistically significant (*P < 0.05, **P < 0.01, ***P < 0.001).

## Results

### Cirsiliol inhibits cell viability, colony formation and wound healing of HCT116 and SW480 cells

**Fig. 1:**
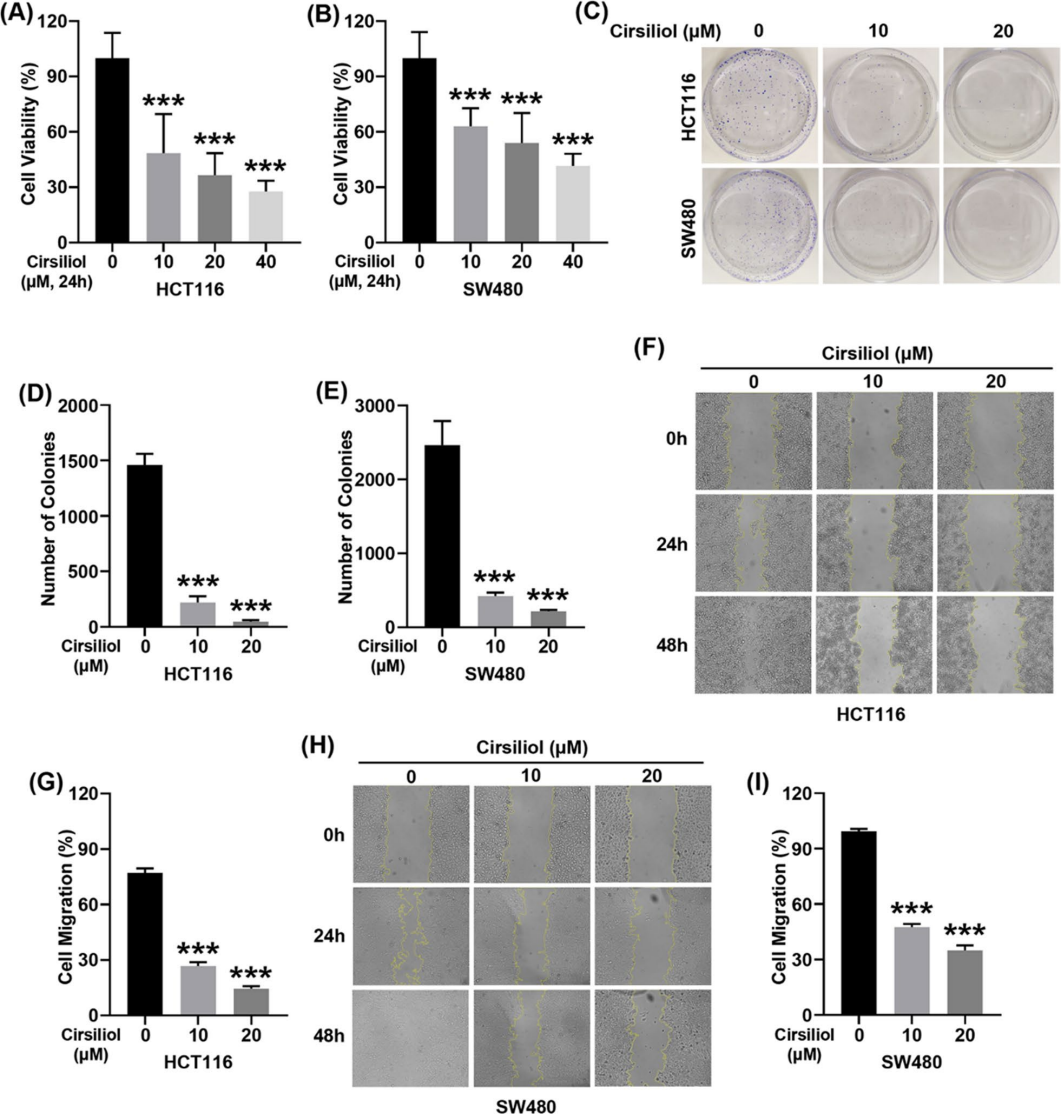
Cirsiliol suppresses cell viability, colony formation and wound healing of colon cancer cells. **A**, **B** CCK assays were carried out to determine cell viability. **C** Images of clone formation assay. **D**, **E** Statistical diagrams of the number of clones of HCT116 (**D**) and SW480 cells (**E**). **F**, **G** Images (**F**) and quantification (**G**) of wound healing assay of HCT116 cells for 48 h. **H**, **I** Photographs (**H**) and quantitative analysis (**I**) of wound healing assay of SW480 cells for 48 h. n = 6 (**A**, **B**), 3 (**C**–**E**) and 4 (**F**–**I**) per group. Data represent means ± SD. ***P < 0.001 vs cirsiliol 0 μM

We first evaluated the anticancer effect of cirsiliol on HCT116 and SW480 cells. Our data showed that cirsiliol suppressed the cell viability in a dose-dependent manner (Fig. 1A, B). Moreover, cirsiliol exhibited the inhibitory effect on cell colony formation (Fig. 1C–E). We also examined the effect of cirsiliol on wound healing ability. Our results revealed that cirsiliol significantly inhibited the wound healing ability of HCT116 and SW480 cells (Fig. 1F–I).

### Cirsiliol suppresses the levels of mitophagy proteins in HCT116 and SW480 cells

**Fig. 2:**
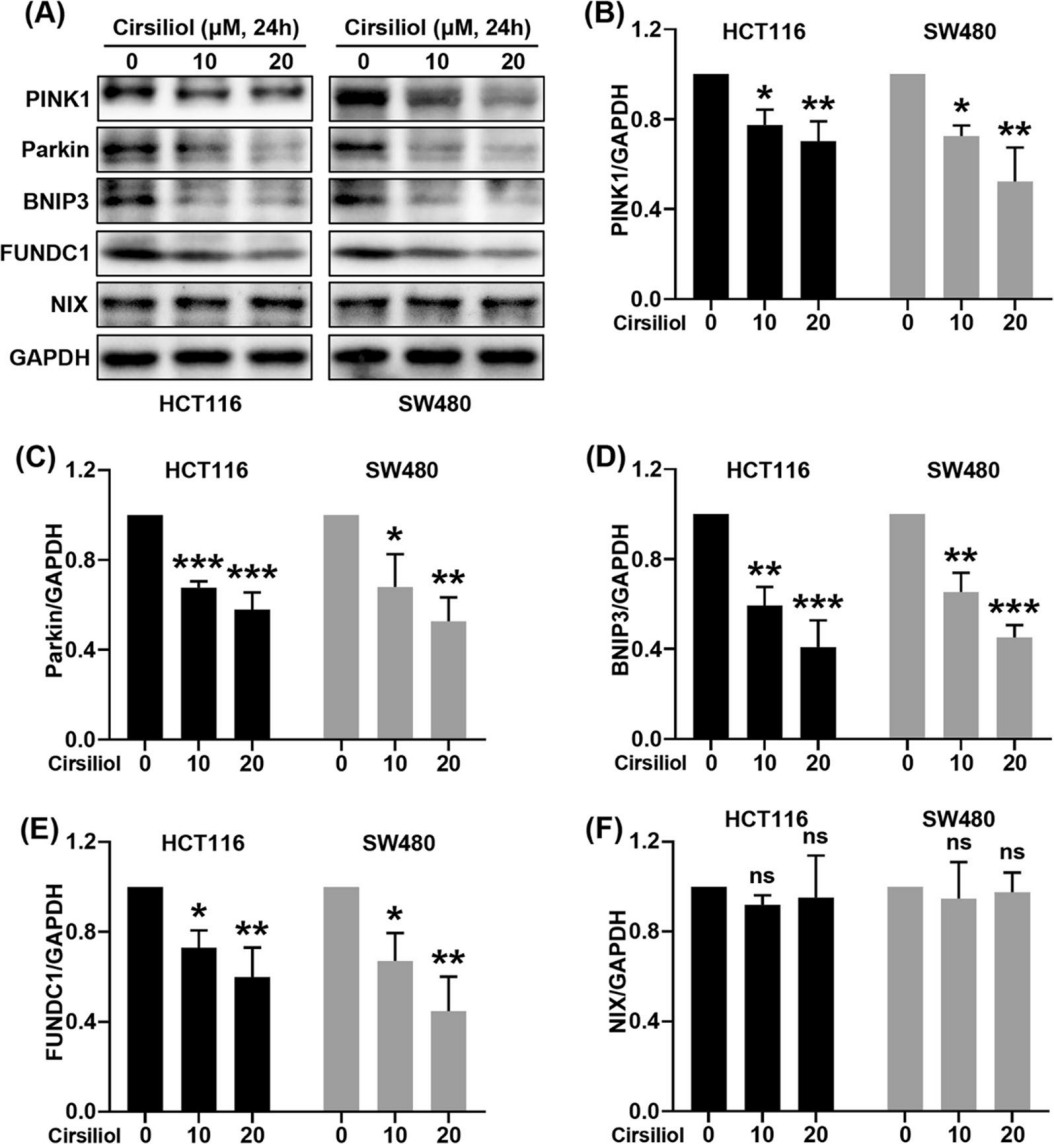
Cirsiliol inhibits the expression of mitophagy proteins in HCT116 and SW480 cells. HCT116 and SW480 cells were treated with cirsiliol for 24 h. **A** The levels of mitophagy proteins were determined by western blot analysis. **B**–**F** Quantitative analysis of western blot for PINK1 (**B**), Parkin (**C**), BNIP3 (**D**), FUNDC1 (**E**), and NIX (**F**). n = 3 per group. Data represent means ± SD. *P < 0.05, **P < 0.01, ***P < 0.001 vs cirsiliol 0 μM

Mitophagy exerts an important role in carcinogenesis, drug resistance and anticancer therapeutics. We checked the key proteins of mitophagy including PINK1, Parkin, BNIP3, FUNDC1, and NIX by western blot analysis in HCT116 and SW480 cells (Fig. 2A). The results showed that cirsiliol concentration-dependently suppressed the expression of PINK1 (Fig. 2B), Parkin (Fig. 2C), BNIP3 (Fig. 2D), and FUNDC1 (Fig. 2E). By contrast, there was no change on NIX expression (Fig. 2F).

### Cirsiliol decreases the mitochondrial membrane potential, promotes the generation of ROS, and disrupts mitochondria network morphology

**Fig. 3:**
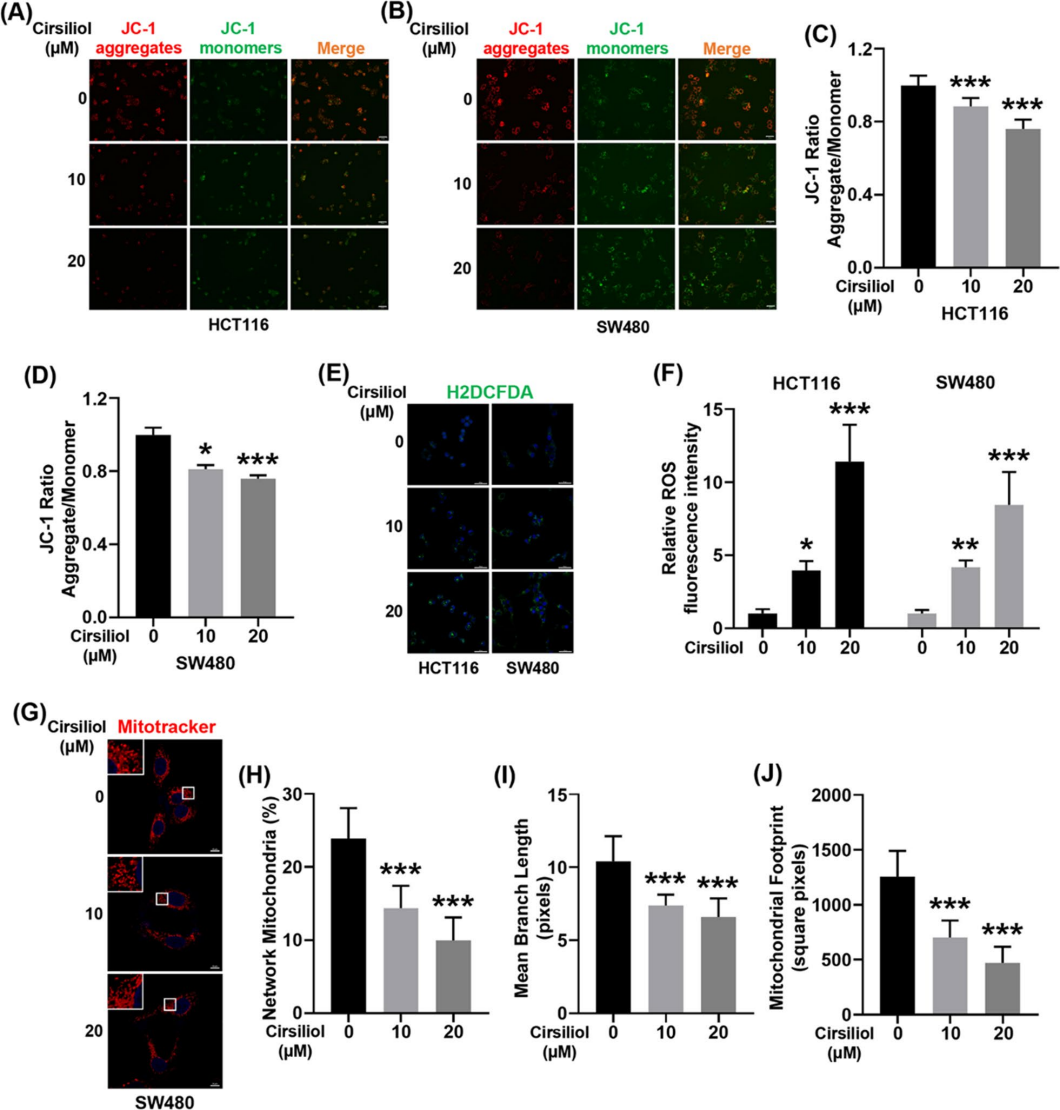
Cirsiliol decreases the mitochondrial membrane potential, promotes the generation of ROS, and disrupts mitochondria network morphology. HCT116 and SW480 cells were treated with cirsiliol for 24 h. **A**, **B** Mitochondrial membrane potential of HCT116 (**A**) and SW480 cells (**B**) was determined by JC-1 staining. Representative fluorescence images of JC-1 aggregate (red) and JC-1 monomer (green) are shown in the Figure. Scale bar: 50 μm. **C**, **D** Quantification of mitochondrial membrane potential (JC-1 aggregate/monomer ratio) for HCT116 (**C**) and SW480 cells (**D**). **E** H2DCFDA staining was performed to detect ROS production for HCT116 and SW480 cells. Scale bar: 50 μm. **F** Quantification of H2DCFDA fluorescence intensity. **G** We performed Mito-Tracker Red staining to detect the mitochondria network morphology of SW480 cells. Representative images were presented in the figure. Scale bar: 10 μm. **H**–**J** MiNA was used to analyze the morphology of the mitochondrial network, including percentage of network mitochondria (**H**), mean branch length (**I**), and mitochondrial footprint (**J**). n = 5 (A-F), and 10 (**G**–**J**) per group. Data represent means ± SD. *P < 0.05, **P < 0.01, ***P < 0.001 vs cirsiliol 0 μM

Mitochondrial damage contributes to the decrease of mitochondrial membrane potential (Δψm). Our results showed that cirsiliol inhibited ∆Ψm in HCT116 and SW480 cells in a dose-dependent manner (Fig. 3A–D). Moreover, decreased ∆Ψm is closely related with elevated ROS production. Therefore, we checked ROS production using H2DCFDA fluorescent probe. We found that cirsiliol increased the ROS production in both HCT116 and SW480 cells in a dose-dependent manner (Fig. 3E, F). Mitochondrial dysfunction alters mitochondrial morphology. Because of scant cytoplasm in HCT116, we just determined the mitochondrial morphology of SW480 cells (Fig. 3G). We found that cirsiliol inhibited the percentage of network mitochondria (Fig. 3H), mean branch length (Fig. 3I), and mitochondrial footprint (Fig. 3J).

### Activation of mitophagy reverses the effects of cirsiliol including the expression of mitophagy proteins, the loss of mitochondrial membrane potential, and ROS production in HCT116 cells

**Fig. 4:**
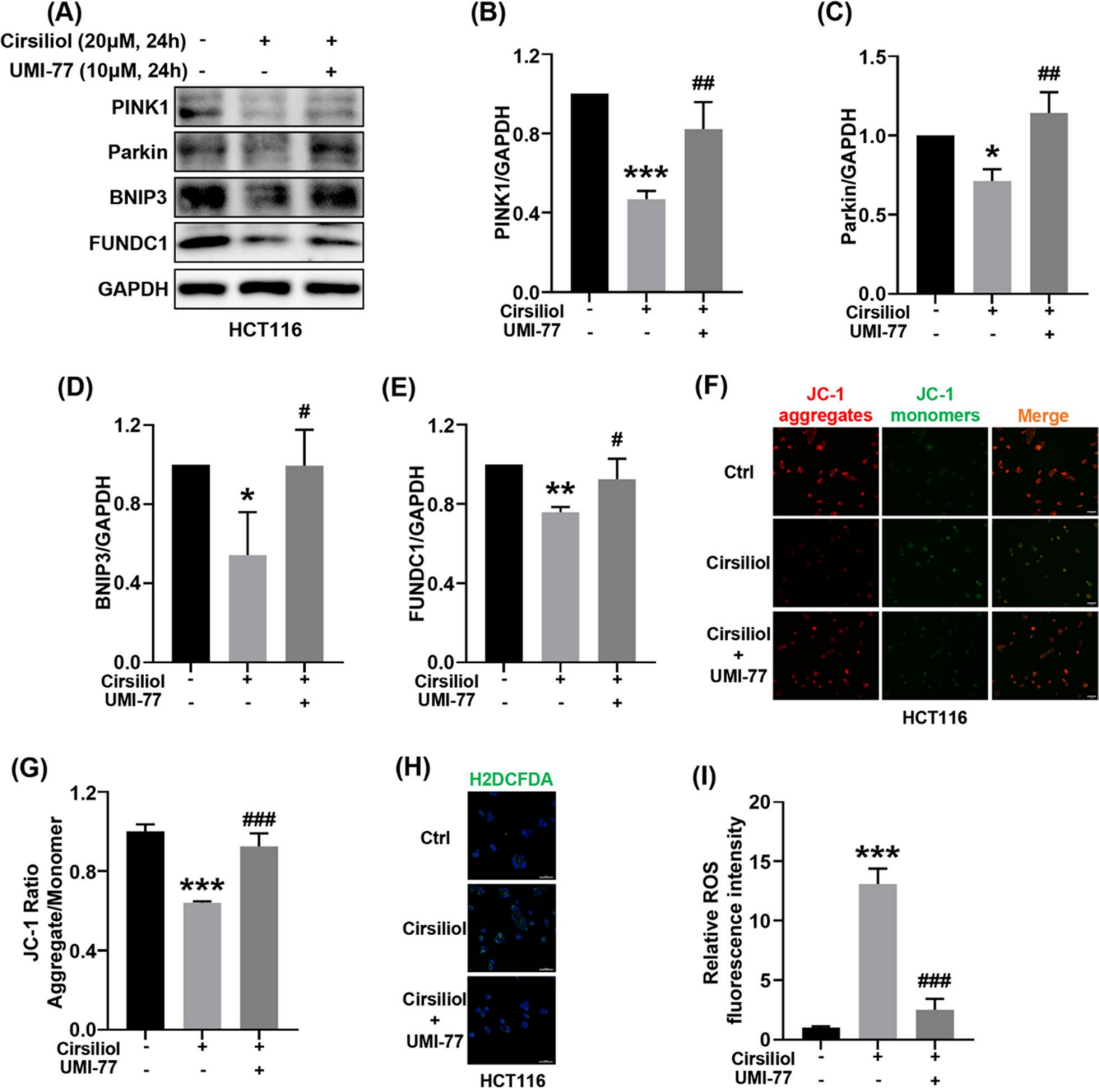
Activation of mitophagy reverses the effects of cirsiliol including the expression of mitophagy proteins, the loss of mitochondrial membrane potential, and ROS production in HCT116 cells. For cirsiliol alone treatment group, HCT116 cells were exposed to 20 μM cirsiliol for 24 h. For UMI-77 group, HCT116 cells were simultaneously treated with 20 μM cirsiliol and 10 μM UMI-77 for 24 h. **A** The levels of mitophagy proteins were determined by western blot analysis. **B**–**E** Quantitative analysis of western blot for PINK1 (**B**), Parkin (**C**), BNIP3 (**D**), FUNDC1 (**E**). **F** Representative images of JC-1 staining. Scale bar: 50 μm. **G** Quantification of the JC-1 fluorescence ratio. **H** Representative images of H2DCFDA staining. Scale bar: 50 μm. **I** Quantification of H2DCFDA fluorescence intensity. n = 3 (**A**–**E**), and 5 (**F**–**I**) per group. Data represent means ± SD. *P < 0.05, **P < 0.01, ***P < 0.001 vs ctrl. ^**#**^*P* < 0.05, ^**##**^*P* < 0.01, ^**###**^*P* < 0.001 vs cirsiliol 20 μM

To confirm the importance of mitophagy in cirsiliol-induced ∆Ψm loss and ROS production, we took advantage of a mitophagy activator, UMI-77 [19]. Because of the prominent effect of 20 μM cirsiliol, this drug concentration was carried out in the following experiments. We first determined the effects of activating mitophagy with the use of UMI-77 in HCT116 cells (Fig. 4A). We found that UMI-77 activated the expression of mitophagy-related proteins suppressed by cirsiliol, including PINK1 (Fig. 4B), Parkin (Fig. 4C), BNIP3 (Fig. 4D), and FUNDC1 (Fig. 4E). We then evaluated whether activation of mitophagy could rescue cirsiliol-induced ∆Ψm loss. The results verified that UMI-77 relieved ∆Ψm loss caused by cirsiliol (Fig. 4F, G). In parallel, UMI-77 extremely attenuated cirsiliol-induced ROS production (Fig. 4H, I).

### Cirsiliol binds with STAT3 and inhibits nuclear translocation of STAT3

**Fig. 5:**
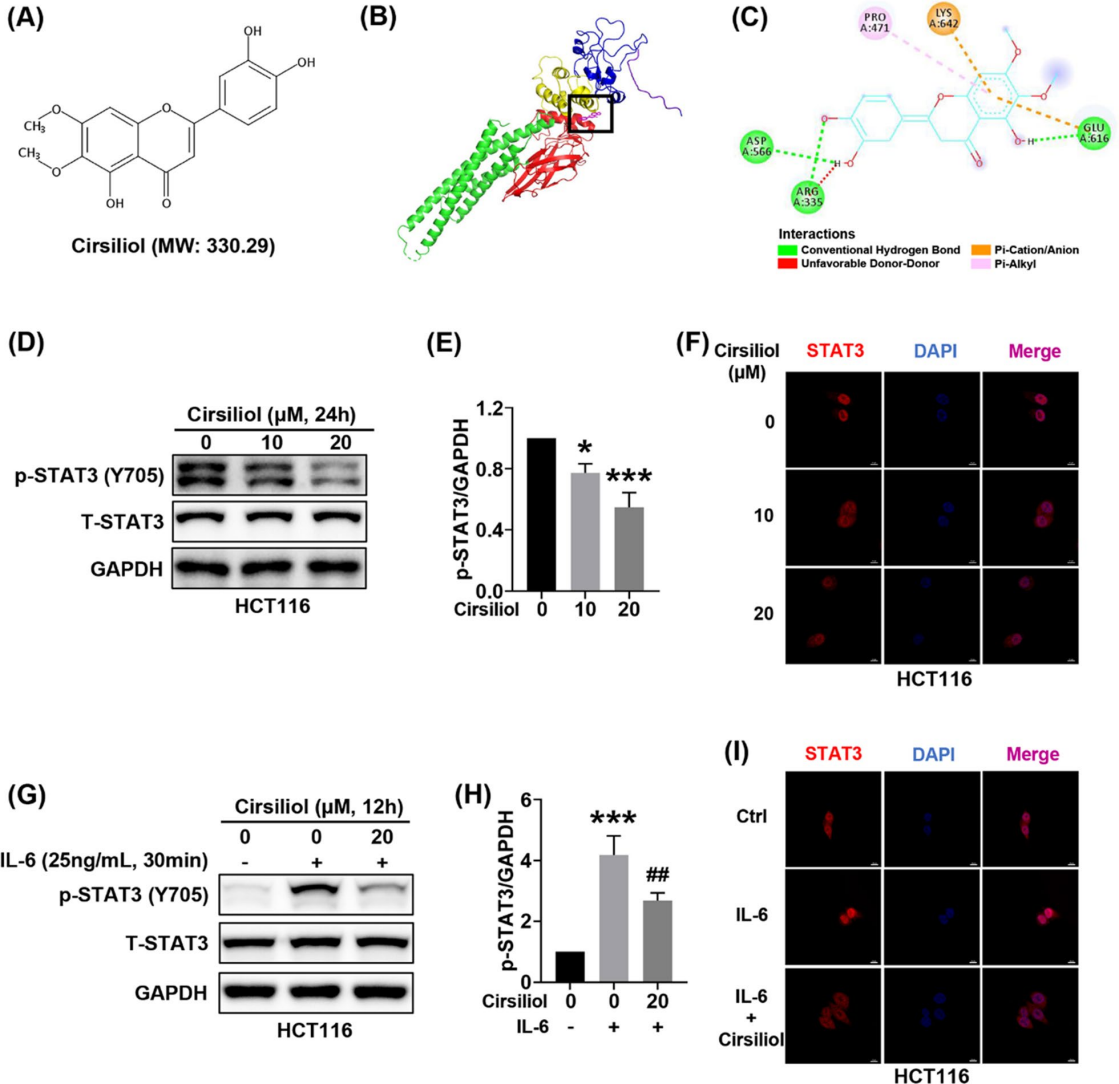
Cirsiliol binds with STAT3 and inhibits STAT3 nuclear translocation. **A** Chemical structure of cirsiliol. **B** Image of molecular docking of cirsiliol and STAT3 (PDB ID: 1BG1). **C** Schematic of the interaction of cirsiliol and STAT3. **D**–**F** HCT116 cells were exposed to cirsiliol for 24 h. Representative western blot images (**D**) and quantitative analysis (**E**) for p-STAT3 and STAT3. Representative immunofluorescence images of STAT3 (**F**). **G**–**I** For IL-6-treated group, HCT116 cells were stimulated by IL-6 (25 ng/ml, 30 min). For cirsiliol treatment, HCT116 cells were treated with cirsiliol (20 μM) for 24 h and stimulated with IL-6 (25 ng/ml) for another 30 min. Representative images of western blot (**G**) and quantitative analysis (**H**) for p-STAT3 and STAT3. Representative immunofluorescence images of STAT3 (**I**). n = 3 (d-e, g-h) per group. Data represent means ± SD. *P < 0.05, **P < 0.01, ***P < 0.001 vs ctrl. ^**#**^*P* < 0.05, ^**##**^*P* < 0.01, ^**###**^*P* < 0.001 vs IL-6 stimulation

Cirsiliol is a natural flavonoid with a molecular weight of 330.29 (Fig. 5A). We applied AutoDock Tools, AutoDock Vina, and Discovery Studio softwares to perform molecular docking between cirsiliol and STAT3 (PDB ID: 1BG1). The results showed that cirsiliol might bind to the SH2 domain of STAT3 (Fig. 5B). The two amino acid residues (Glu-616 and Lys-642) in the STAT3 SH2 domain formed hydrogen bonds and pi-cation/anion interactions with cirsiliol (Fig. 5C). In addition, we found that cirsiliol suppressed the activation of STAT3 in a concentration-dependent manner, with no significant inhibition of total STAT3 (Fig. 5D, E). Subsequently, we examined the nuclear translocation of STAT3 by immunofluorescence. Our results indicated that cirsiliol inhibited STAT3 nuclear translocation (Fig. 5F). IL-6 has been shown to contribute to STAT3 activation in colon cancer. Our results showed that cirsiliol prevented STAT3 activation and nuclear translocation induced by IL-6 (Fig. 5G–I).

### Overexpression of STAT3 promotes mitophagy suppressed by cirsiliol in HCT116 cells

**Fig. 6:**
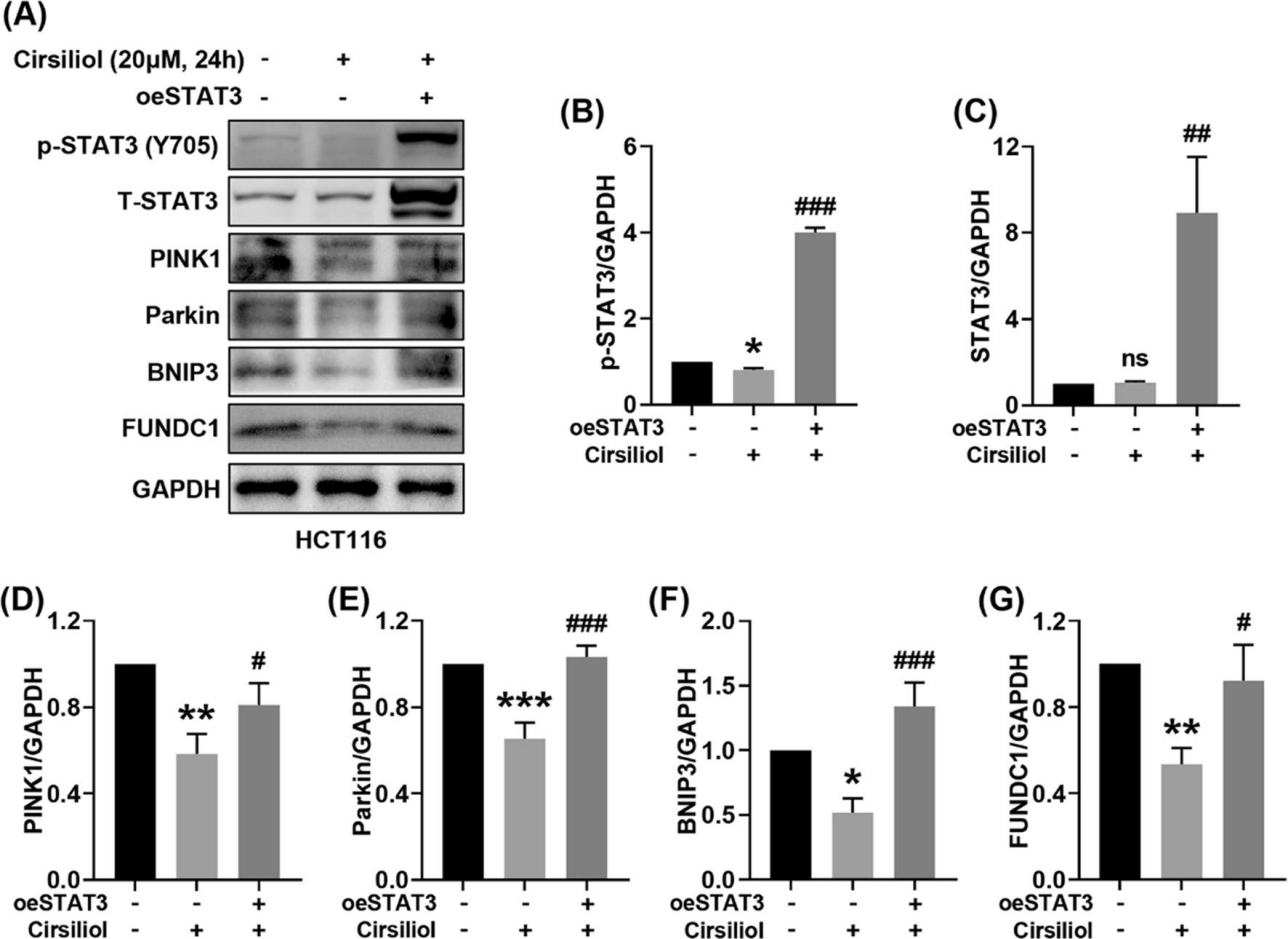
Overexpression of STAT3 promotes mitophagy suppressed by cirsiliol in HCT116 cells. HCT116 cells were transfected with control plasmid or STAT3 overexpression plasmid (oeSTAT3) for 48 h and incubated with cirsiliol for another 24 h. **A** Representative western blot images of p-STAT3, total STAT3 and mitophagy proteins. **B**–**G** Quantitation of p-STAT3 (**B**), STAT3 (**C**), PINK1 (**D**), Parkin (**E**), BNIP3 (**F**), FUNDC1 (**G**). n = 3 per group. Data represent means ± SD. *P < 0.05, **P < 0.01, ***P < 0.001 vs ctrl. ^**#**^*P* < 0.05, ^**##**^*P* < 0.01, ^**###**^*P* < 0.001 vs cirsiliol without oeSTAT3

To further confirm the role of STAT3 in the regulation of mitophagy by cirsiliol, we utilized STAT3 overexpression plasmid (oeSTAT3) to overexpress STAT3 in HCT116 cells and checked the levels of mitophagy proteins as well as STAT3 (Fig. 6A). Our results showed that cirsiliol decreased the level of phosphorylated STAT3 with no significant inhibition of total STAT3, and overexpression of STAT3 remarkably increased the levels of p-STAT3 and total STAT3 (Fig. 6B, C). Of note, overexpression of STAT3 increased the expression of mitophagy proteins suppressed by cirsiliol, including PINK1 (Fig. 6D), Parkin (Fig. 6E), BNIP3 (Fig. 6F), and FUNDC1 (Fig. 6G).

## Discussion

CC is the leading cause of tumor-related death in many countries [20]. Currently, surgery combined with chemotherapy is the main treatment of colon cancer [21]. The current chemotherapies for colon cancer often offer limited help because cancer cells are relatively resistant to chemotherapy and promote tumor relapse and metastasis [22]. Therefore, in-depth understanding the pathogenesis of colon cancer is of great urgency.

Mitochondria are important organelles and considered “powerhouses of the cells”. Mitochondrial function serves an essential role in cancer cell survival and tumor progression, and recent literatures have targeted mitochondria as a new target for cancer chemotherapy [23]. Mitophagy is a selective autophagy of mitochondria that responses to mitochondrial dysfunction promotes mitochondrial quality control [24]. Ubiquitin‐mediated mitophagy and receptor‐mediated mitophagy are widely studied mitophagy. Representative ubiquitin‐mediated mitophagy is PINK1/Parkin-mediated mitophagy, which is also known as the classical mitophagy. FUNDC1, BNIP3, and NIX have been found to participate in receptor‐mediated mitophagy with LC3-interacting region. PINK1 [25], Parkin [26], BNIP3 [27], NIX [28], and FUNDC1 [29] have been reported to facilitate tumor progression and recurrence. In contrast, several studies have shown that mitophagy suppresses tumorigenesis [30, 31]. Of note, mitophagy may play different roles in different pathological stages of tumor. We speculated that mitophagy inhibited the accumulation of dysfunctional mitochondria and reduced tumorigenesis in normal cells; and in tumor cells, mitophagy can remove damaged mitochondria, maintain the dynamic balance of mitochondrial function, and promote tumor progression. Certainly, mitophagy may have a different role in different types of tumors. Our results showed that natural compound cirsiliol suppressed the expression of mitophagy proteins in dose-dependent manner. Δψm is an effective biomarker to evaluate the function of the mitochondria. The depletion of Δψm is an early event of the apoptotic process [32]. Our results showed that cirsilol inhibited Δψm in a concentration-dependent manner. Mitochondrial ROS generation has been considered to be dependent on mitochondrial membrane potential [33], which is consistent our results. Furthermore, the application of mitophagy activator rescued loss of Δψm and ROS production caused by cirsiliol. Thus, this suggested that mitophagy was benefit for the development of colon cancer and cirsiliol induced the apoptosis of tumor through inhibiting mitophagy.

Inflammation is the seventh major hallmark of malignant tumors and is related with cancer initiation and progression, especially colon cancer [34]. In more recent studies, it is reported that mitophagy is closely related with infection and inflammatory disease [35, 36]. However, the link between inflammation and mitophagy in cancer development is largely undefined. STAT3 is widely activated in multiple human cancers and has been an attractive target for cancer therapeutic [37]. Numerous studies have shown that STAT3 serves a significant role in multiple aspects, including tumor invasion, migration, metastasis, and angiogenesis [38]. In agreement with above studies, our previous study indicated that STAT3 is an important target for colon cancer [39]. STAT3 is known as a transcription factor and phosphorylation of STAT3 at Tyr705, which contributes to STAT3 dimerization, nuclear translocation and transactivation of target genes [40]. It is known that IL-6 strongly activates STAT3. We found that cirsiliol could dose-dependently inhibited the activation and nuclear translocation of STAT3. IL-6-induced STAT3 phosphorylation and nuclear translocation can also be inhibited by cirsiliol. Using molecular docking technique, we found cirsiliol could bind with the SH2 domain of STAT3, which is related with activation, dimerization, and nuclear translocation of STAT3 [41]. Overexpression of STAT3 activated mitophagy suppressed by cirsiliol. Therefore, cirsiliol could regulate mitophagy through inhibiting STAT3 activation. Previous studies [42, 43] show that STAT3, as a transcription factor, promotes the expression of BNIP3 in concanavalin a-induced hepatitis and glioblastoma cells. Our results suggested that other mitophagy proteins might also be the potential target genes in colon cancer.

In recent years, natural compounds are reported to have anticancer effects. Strengths of natural compounds for treatment with tumor are the higher efficacy and lower toxicity. Cirsiliol is one of the flavonoids compounds and have been reported have a therapeutic efficacy in non-small cell lung cancer cell lines [10], malignant melanoma cells [7], human leukemia cell lines [44], esophageal squamous cell carcinoma [9], and so on. Our results first showed that cirsiliol inhibited cell viability, colony formation and wound healing of colon cancer cells. Lim et al. [8] have pointed out that cirsiliol can inhibit STAT3 activation induced by IL-6 and the results were further confirmed in our study through molecular docking.

## Conclusions

In conclusion, we first found that cirsiliol inhibited cell viability, colony formation and wound healing of colon cancer cells. Cirsiliol exhibited the suppression of mitophagy proteins including PINK1, Parkin, BNIP3, and FUNDC1, leading to the reduction of mitochondrial membrane potential and ROS production. More importantly, we found that cirsiliol could bind to SH2 domain of STAT3 and inhibit STAT3 activation and nuclear translocation. Overexpression of STAT3 activated mitophagy suppressed by cirsiliol. Therefore, cirsiliol is a potential candidate for the treatment of colon cancer and works by inhibiting mitophagy via STAT3 signaling.

## Data Availability

The datasets used and/or analyzed during the current study are available from the corresponding author on reasonable request.

## Abbreviations

Δψm: Mitochondrial membrane potential
ANOVA: Analysis of variance
CC: Colon cancer
DMSO: Dimethyl sulfoxide
PDB: Protein data bank
ROS: Reactive oxygen species level
STAT3: Signal transducer and activator of transcription 3
